# Contribution of Eye-Tracking to Study Cognitive Impairments Among Clinical Populations

**DOI:** 10.3389/fpsyg.2021.590986

**Published:** 2021-06-07

**Authors:** Alexandra Wolf, Kazuo Ueda

**Affiliations:** ^1^JSPS International Research Fellow, Research Center for Applied Perceptual Science, Kyushu University, Fukuoka, Japan; ^2^Unit of Perceptual Psychology, Dept. Human Science, Research Center for Applied Perceptual Science, Division of Auditory and Visual Perception Research, Research and Development Center for Five-Sense Devices, Kyushu University, Fukuoka, Japan

**Keywords:** clinical research, cognitive impairments, consumer science, eye-tracking, neuromarketing, translational practice

## Abstract

In the field of psychology, the merge of decision-theory and neuroscientific methods produces an array of scientifically recognized paradigms. For example, by exploring consumer’s eye-movement behavior, researchers aim to deepen the understanding of how patterns of retinal activation are being meaningfully transformed into visual experiences and connected with specific reactions (e.g., purchase). Notably, eye-movements provide knowledge of one’s homeostatic balance and gatekeep information that shape decisions. Hence, vision science investigates the quality of observed environments determined under various experimental conditions. Moreover, it answers questions on how human process visual stimuli and use gained information for a successful strategy to achieve certain goals. While capturing cognitive states with the support of the eye-trackers progresses at a relatively fast pace in decision-making research, measuring the visual performance of real-life tasks, which require complex cognitive skills, is tentatively translated into clinical experiments. Nevertheless, the potential of the human eye as a highly valuable source of biomarkers has been underlined. In this article, we aim to draw readers attention to decision-making experimental paradigms supported with eye-tracking technology among clinical populations. Such interdisciplinary approach may become an important component that will (i) help in objectively illustrating patient’s models of beliefs and values, (ii) support clinical interventions, and (iii) contribute to health services. It is possible that shortly, eye-movement data from decision-making experiments will grant the scientific community a greater understanding of mechanisms underlining mental states and consumption practices that medical professionals consider as obsessions, disorders or addiction.

*To consider the state of the decision-maker translated by the beholder’s eyes is our goal*

## Introduction

Consumers make decisions at their own pace. Their choices are significantly influenced by personal preferences, situational context of the decision, such as presence of time pressure and size of the opportunity set (a number of given alternatives) as well as the environment of the point of purchase (Baldwin et al., 2012; Venkatraman et al., 2012; Berčík et al., 2016; Spence et al., 2016; Cherubino et al., 2019; Lin et al., 2019; Wolf et al., 2019; Vriens et al., 2020). Importantly, consumers are usually not aware of the steps of simplifying the decision processes by eliminating (ignoring) some information and paying attention (giving more time) to certain, considered options. Hence, technological advances that enable to isolate key processes, which underlay individuals’ preferences and reactions (e.g., buying behavior), attain increasing attention of the media, user analysts and researchers (Hubert and Kenning, 2008; Chynal et al., 2016; Oliveira et al., 2016; Gidlöf et al., 2017; Touchette and Lee, 2017; Spence, 2019).

The application of neurophysiological tools recording brain measures (e.g., electrical brain activity, functional near-infrared spectroscopy, BOLD-contrast imaging used in functional magnetic resonance imaging, fMRI) and non-brain measures (e.g., electrodermal response, heart rate, eye-tracking), as adjuvant instruments to behavioral data in marketing research, is not a new concept (Levy et al., 2011; Berčík et al., 2016; Cherubino et al., 2019). Studies of eye-movements through direct observations were conducted already in the 1800s. An early form of an eye-tracker was built by Edmund Huey in 1908 and first non-intrusive eye-tracker was constructed by a pioneer in experimental educational psychology — Guy Thomas Buswell, known for groundbreaking investigations on recording and analyzing subjects’ eye movements (Buswell, 1935). Buswell’s results indicated that observers often fixated on the same spatial locations in an image, but not necessarily in the same temporal order. Moreover, the viewers’ eyes tended to focus on foreground elements (e.g., faces and people) rather than background elements (e.g., clouds or foliage). In 1945, Brandt, 1945 published a general analysis of eye movement patterns of participants, who looked at advertisements. Similarly to Buswell, Brandt concluded that there are noticeable, individual differences in eye movements, but in general, these behaviors are similar enough in order to formulate “psychological laws” (see Babcock et al., n.d.)

The work on the relationship between eye-movements and the sequence of thought processes has been greatly extended by a Russian psychologist Alfred Lukyanovich Yarbus, who believed that the viewers’ eyes were directed toward areas of the stimulus that were “useful or essential” to perception. Recorded eye-movements of examining Ilya Repin’s painting *“An Unexpected Visitor*,*”* provided key findings on the substantial influence of experimental task on viewer’s eye-movements (kindly refer to the book Yarbus, 1967, which is often quoted among researchers investigating the relationship between fixations and interest, Mackworth and Bruner, 1970; Noton and Stark, 1971; Walker Smith et al., 1977; Hayhoe and Ballard, 2005; Unema et al., 2005; Castelhano et al., 2009; Greene et al., 2012; Borji and Itti, 2014; Martinez-Conde and Macknik, 2015; Boisvert and Bruce, 2016; Smith et al., 2018).

*“(…) Eye movement reflects the human thought processes; so the observer’s thought may be followed to some extent from records of eye movement (the thought accompanying the examination of the particular object). It is easy to determine from these records which elements attract the observer’s eye (and, consequently, his thought), in what order, and how often.”* (Yarbus, 1967, *p. 190)*

Until now, human eye-positions receive much attention to gain objective insights into how consumers sample information, make decisions under different task-instructions or mind-wander, where the slightest change in gaze allocation reflects a shift in information-prioritization (Yarbus, 1967; Baron-Cohen et al., 1997; Najemnik and Geisler, 2005, 2009; Castelhano et al., 2009; Tatler et al., 2010; Morii and Sakagami, 2015; Boisvert and Bruce, 2016).

Principally, in order to find the optimal outcome, decisional processes such as (i) assessment and formation of preferences, (ii) selection and execution of action(s), (iii) experiencing the outcome, are all orchestrated in human brain systems. A combination of eye-tracking methodology and decision-making (high-level information processing) paradigms can result in understanding one’s cognitive and affective mental state and explain reactions in a real-world context (Bogacz et al., 2006; Bogacz, 2007; Paulus, 2007; Cocker et al., 2012). Moreover, there is growing evidence that decision-making and homeostatic processing are inextricably linked, and that cognitive impairments (e.g., disturbances of memory, attention deficits and difficulty in problem-solving) among clinical populations cannot be understood without the reference to the performance of independent living skills (Brand et al., 2005; Paulus, 2007; Bachman et al., 2010).

Since eye-tracking instruments objectively reflect action selection, based on decision maker’s attention, information processing capabilities, and motivated cognition processes (Deubel and Schneider, 1996; Ernst and Paulus, 2005; Simion and Shimojo, 2006; Glaholt and Reingold, 2009; Markkula, 2015; van der Laan et al., 2015; Gerbella et al., 2017; Onuma et al., 2017; Wolf et al., 2018, 2019), it should not be overlooked that relatively cost-effective and non-invasive eye-tracking devices are becoming vital instruments exposing the mechanism of cognitive and perceptual disturbances among clinical populations (Tseng et al., 2013; Türkan et al., 2016; Sawada et al., 2017; Morita et al., 2020; Wolf et al., 2021).

## Visible Impairments in Information Processing

Longitudinal persistence of exhibited deficits in intellectual functioning, i.e., attention, memory and executive function, suggest that information processing abnormalities are related to the core of a vast number of mental disorders. For example, Autism spectrum disorder (ASD) adults and children have been reported to spend less time looking at silent features of face stimuli (mouth, nose, and eyes regions) and more time at non-social stimuli than healthy controls (HC) (Corden et al., 2008; Frazier et al., 2016). Furthermore, while viewing video clips, young ASD adults have been reported to spend more time gazing at objects (instead of people). These observations support a poorer social adjustment among ASD patients, where by fixating on non-social stimuli, ASD patients are predestinated to miss socially important cues (Klin et al., 2002).

In the context of eating disorder group, it has been suggested that the majority of studies investigated the attentional bias toward disorder-relevant stimuli, which was based on reaction times that measured attention indirectly, i.e., without revealing its time course (Shafran et al., 2007; Sperling et al., 2017; Kerr-Gaffney et al., 2019). With the use of eye-tracking technology, however, it is possible to measure attention directly, where for example measurements of the overall viewing time, scan-path length, number of saccades, and the differentiation between early and late stages of attentional processing are achievable (Giel et al., 2011). Jansen and colleagues found that participants with eating disorders, when viewing photos of their own bodies, focused less on their self-defined *beautiful* body parts and more on their *ugly* parts than the HC group did (i.e., inspection of the ugly body parts was given priority). However, when viewing pictures of others, the eating disorder group showed the opposite pattern (Jansen et al., 2005).

Recent studies, which investigated food and non-food stimuli under free-viewing task, report that both groups: binge eating disorder (BED) and HC, tend to fixate their gaze longer on non-food images. However, when comparing the total viewing time for food pictures, those with BED visually attended to them for a longer time duration than HC (Schag et al., 2013; Schmidt et al., 2016; Sperling et al., 2017). Interestingly, Baldowski and his team reports that individuals with night eating syndrome show an initial orienting bias toward food stimuli over non-food images (Baldofski et al., 2018).

It has been reported that individuals with social anxiety disorder show a quicker attentional disengagement from the eyes, in line with the vigilance-avoidance theory of attention (Horley et al., 2003, 2004). In two experiments reported by Boll and colleagues, patients with social phobia and HC were compared regarding their gaze behavior toward displayed photos of human faces (Boll et al., 2016). The results from performed paradigms (emotion classification paradigm and gaze-cueing paradigm) indicate that in comparison to HC, patients reflexively orient their attention toward the eyes of emotional faces in the emotion classification paradigm. This initial hypervigilance for the eye region can be observed at very early attentional stages and persists for a longer duration of time. Moreover, Boll and colleagues reported that individuals with social phobia exhibit a delayed attentional orienting into the direction of eye gaze. This observation may suggest a differential time course of eye gaze processing in individuals with social phobia and HC.

The fundamental issue of eye behavior reflecting attention and its deficits (Yarbus, 1967; Braff, 1993; Rommelse et al., 2008; DeAngelus and Pelz, 2009; Borji and Itti, 2014) attracts the scientific community as a measurable indicator of one’s sequence of thoughts (Bird et al., 2012; Thakkar et al., 2018; Wolf et al., 2018, 2019). Therefore, we put forward the statement that eye-movement measurements gathered from visual information-processing paradigms are already supporting the development of integrated eye movement scores (kindly refer to Morita et al., 2017; Obyedkov et al., 2019).

Information processing deficits are an important area of investigation focusing on identifying the key features of life-impoverishing disorders that may allow efficient early intervention and support the discovery of preventive treatments. Moreover, for the reason that almost every area of the brain plays a role related to the ocular motor control system, the implementation of eye-tracking technology to cognitively informative tasks, has proven to be a fruitful approach in uncovering human information processes (Jarodzka et al., 2010; Glaholt and Reingold, 2011; Lai et al., 2013; Pärnamets et al., 2015; Roberts et al., 2018). However, mental health professionals avoid capturing eye-data through tasks that may have “too unusual demands” or could be labeled as “too complicated.” This may explain why real-life oriented decision-making paradigms are hesitantly used to discover the mechanisms that underlie non-optimal (non-homeostatic) behavior among clinical populations.

### Loss of Social Autonomy Due to Cognitive Impairments

Unequivocally, scientists need to be thoughtful about patients, who suffer from psychiatric disorders such as schizotypal personality disorder (STPD), schizophrenia (SZ), major depressive disorder (MDD), bipolar disorder (BD), and ASD (which entails a spectrum of disorders, namely autism, Asperger’s disorder, and pervasive developmental disorder-not otherwise specified, PDD-NOS). While experiencing various cognitive impairments, clinical populations may face disinterest in social contact, discomfort in interpersonal situations, decreased ability to feel pleasure (physical anhedonia), and/or diminished experience of reward (Cocker et al., 2012). Notably, some patients are being unable to independently execute instrumental activities of daily living, e.g., managing money, commuting, preparing meals, shopping. For example, BP patients may excessively involve in activities with high potential for painful consequences (e.g., engaging in unrestrained buying sprees or impulsive business investments), being therefore at a higher risk of gambling and compulsive spending. Overall, clinical populations are either omitted as decision-makers, facing a loss of social autonomy (Mottron et al., 2006; Sablier et al., 2009), or taken advantage of due to the inability to control their consumption practices (Jones et al., 2015).

Therefore, it is crucial to identify the relationship between cognitive deficits and specific activities among people with psychiatric diseases. In order to do so, few research groups considered designing experimental paradigms to resemble a real-life tasks (Hamera and Brown, 2000; Rempfer et al., 2003; Zayat et al., 2011; Laloyaux et al., 2012, 2013). In 2002 Hamera and colleagues conducted a grocery shopping skill test, measuring the performance of shopping ability, which requires complex cognitive skills (Hamera et al., 2002). In this particular study, the performance of two groups has been compared, i.e., individuals with significant cognitive impairments (SZ and BD patients) and a normative population of HC. In particular, the researchers reported that individuals with SZ and BD took longer time in task completion. Besides, the group with significant cognitive impairments showed higher redundancy in performing the shopping task than the healthy control group.

Moreover, in another shopping task investigating buying performance, where participants were required to purchase grocery store items from a shopping list, Laloyaux et al. (2013) significantly differentiated BD patients from HC for two variables, namely (1) the total time to complete the shopping and (2) the mean time spent to consult the grocery list. The same research group reported that the performance of a shopping task correlated significantly with individual’s cognitive functioning (i.e., processing speed, verbal episodic memory, planning, and cognitive flexibility) and with clinical variables, such as duration of illness and functioning in an authentic life context (Laloyaux et al., 2013).

Described examples provide evidences of reliability and validity of context-based tests related to purchase intentions and shopping performance measurement (Manee, 1997; Hamera and Brown, 2000; Rempfer et al., 2003). Also, current advances in eye-tracking technology and data analysis (McGrath et al., 2018; Zommara et al., 2018) allow the scientific community to conduct follow-up experiments (and/or build new ones) in order to provide a deeper knowledge concerning how cognitive deficits impact clinical population in a real-world functioning context.

Future decision-making paradigms may provide promising reaction-derived data, disclosing repressed feelings, unconscious reactions/habits. This may help to clarify the basis of high heritability, associated with psychiatric disorders, and promoting transdiagnostic research. Moreover, it may refine the diagnosis procedure, which is highly challenging, especially in heterogeneous disorders such as schizophrenia (see Koychev et al., 2011). The implementation of eye-tracking methodology appears to be a straightforward undertaking. Through the detected position of the viewer’s pupil, gaze points can be easily determined and further analyzed with the use of mathematical algorithms. Hence, the implementation of eye-tracking technology fits perfectly the aim to non-invasively disclose valuable information regarding individual’s behavior, which cannot be as precisely discovered through written nor verbal approaches (Riby and Hancock, 2008; Dewhurst et al., 2012; Chawarska et al., 2013; Maruta et al., 2014; Diwakar et al., 2015; Fujioka et al., 2016; Crawford et al., 2017; Oyama et al., 2019).

### Revealing One’s Moment-by-Moment Focus of Attention

It has been stated that eye-movement technology provides a moment-by-moment measure of the focus of attention and reveals what cognitive strategies (interplay of top-down and bottom-up processing) are employed to solve a particular task (Eckstein et al., 2017). In the past years, experts have used *pupil dilation* data to reveal for example the subjective difficulty of cognitive tasks and their intensity (mental effort). The choice of this parameter has been supported with a common knowledge that pupil dilation provides a window into the brain’s locus coeruleus-norepinephrine (LC-NE) system (Laeng et al., 2012; Burkhouse et al., 2015; Chevalier et al., 2015; Bast et al., 2020). Neurophysiological findings provide significant insights to the meaning of pupillary responses for mental activity. Given that pupillary responses can be easily measured in a non-invasive manner in each stage of life, and can occur in the absence of conscious processes, they constitute a promising tool for the study of preverbal (e.g., infants) and non-verbal participants.

Furthermore, *spontaneous eyeblink rate* (sEBR), as a proxy of dopaminergic activity (Jongkees and Colzato, 2016; Eckstein et al., 2017; Gotlieb et al., 2021) has been used to study cognition, interest, and predict stress levels in humans (Chermahini and Hommel, 2010; DeYoung, 2013). Since spontaneous blinks are uniquely different from voluntary and reflexive eye-blinks, they represent a range of information processing functions spanning attention and working memory (Müller et al., 2007; Schumacher et al., 2013; Groman et al., 2014). For example, humans subjected to stressful stimuli (through social and emotional recollection tests) were reported to exhibit an increase in spontaneous blink rate. Several studies reported that increased eyeblink rate reflected an increasing level of fatigue (Beatty, 1982; Borghini et al., 2014; Gergelyfi et al., 2015). On the other hand, spontaneous blink rate has been found to decrease when the subjects are most attentive in performing demanding tasks associated with memory operations and attentive behaviors (see Hirokawa et al., 2004; Brefczynski-Lewis et al., 2011; Paprocki and Lenskiy, 2017). Moreover, eye blink parameters enable to investigate the influence of specific stressors that initiate emotional anxiety or neurological levels of arousal (van de Groep et al., 2017).

**FIGURE 1:**
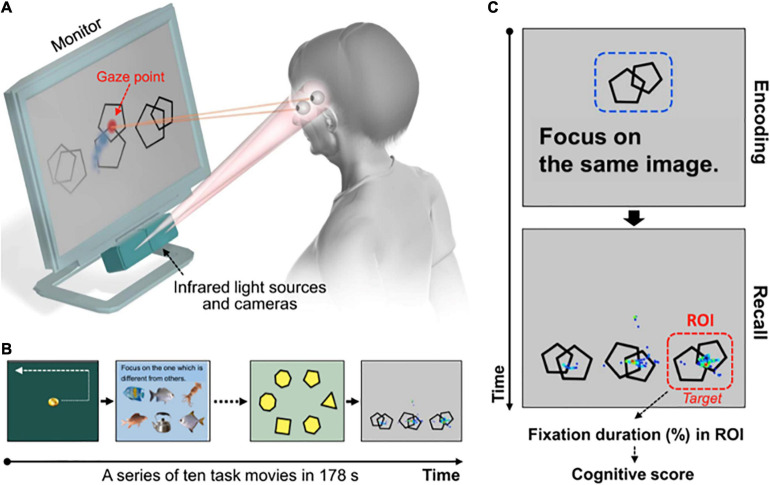
Rapid cognitive assessment using an eye-tracking system and tasks, obtained from Oyama et al. (2019). **(A)** Participant’s gaze points are recorded. **(B)** Ten tasks are displayed one by one on the monitor. **(C)** An example of a working memory task and representative gaze plots with a duration-based heatmap obtained from a control subject. Gaze plots represent the location and time spent looking at the objects. A cue object (e.g., a set of double pentagons) is presented for 10 s (*encoding phase*), followed by three distinct objects with the same one as the cue object (right bottom on the monitor, *target*). In the next phase (*recall*) the participant is asked to remember and gaze at the target object. Fixation duration within the region of interest (ROI) set on the target object is used to obtain the cognitive score.

*Area of interest* (AOI) that is not categorized as a gaze metric by itself but defines regions by which gaze metrics may be calculated, has been reported to help in studying reasoning on account of integrating several pieces of information and reflecting the process of comparing specific locations of presented stimuli (Dewhurst et al., 2012; Hunter et al., 2020). Recently, to assess cognitive function using high-performance eye-tracking technology, Oyama and colleagues developed a novel cognitive assessment tool (Oyama et al., 2019), which has been reported as brief and practical since the subject simply views a series of short movies and pictures displayed on a monitor. In each task, multiple stimuli, including the target image (correct answer) and non-target images (distractors), are presented on the monitor. The subject is instructed to identify and focus her gaze on the correct answer. The AOI is set on the correct answer, and the cognitive score is determined from the eye-movement data by measuring the fixation time on the region of the correct answer (kindly refer to Figure 1). Oyama and colleagues concluded that the cognitive score correlated well with the scores from neuropsychological tests, showing an outstanding diagnostic performance in detecting patients with mild cognitive impairment (MCI) and dementia (Oyama et al., 2019).

Next, *scanning patterns* have been widely covered in the scientific literature, giving insight into one’s exploration behavior, directly related to the nature of the task (Kojima et al., 1992; Bestelmeyer et al., 2006; Castelhano et al., 2009; Risko et al., 2012; Sprenger et al., 2013; Helo et al., 2014; Dowiasch et al., 2016; Yazdan-Shahmorad et al., 2020). Recently, exercising the differences between subjects’ visual scanning patterns Chung et al. (2018) could disambiguate bipolar and unipolar patients with high accuracy (Chung et al., 2018). Since misdiagnosing BD as MDD is relatively common, the introduction of biomarkers to improve diagnostic accuracy, in the earliest course of the illness, plays an important role in the clinical field. With the use of recurrent neural network (RNN), the differences between fixation sequences provided a classifier that disambiguated BD from MDD patients with a remarkably high accuracy.

Finally, *gaze duration* (viewing time) that reflects (i) the quality of the available visual information, (ii) the contextual background with which objects can be recognized and understood, (iii) individual goals of the observer, and (iv) information-search strategies (Underwood et al., 2008; Najemnik and Geisler, 2009; Schotter et al., 2010; Foulsham and Kingstone, 2011, 2013; Pierce et al., 2016; Roux et al., 2016; Saito et al., 2017) has been pointed out to be a useful index of the extent of information processing (Bird et al., 2012; Wolf et al., 2018, 2019). Moreover, Wolf and colleagues suggested that uncertainty can prolong the effort of visual processing (Wolf et al., 2019).

Taken together, data commonly captured by eye-trackers such as (i) eye opening and closure (e.g., blink duration, blink frequency), (ii) gaze parameters (e.g., number of fixations, saccades, viewing time), (iii) pupil properties (e.g., pupil dilation, pupil size), give valuable insights into how a viewer (un)consciously filters information, undertakes the decision strategy, and determines the subjective hierarchy of perception. Moreover, human eye-movements have illustrated valuable insights regarding the information processing patterns, translated into attentional landscapes, fixation sequences, or heat maps, and served as measurable outputs of the extent of undergoing cognitive processes (Bird et al., 2012; Pärnamets et al., 2016; Zommara et al., 2018). In conclusion, recordings of the eye-movements provide answers to important questions of when and how visual information is being captured and processed in scientifically controlled as well as ecologically valid environments (for outstanding work on implementing ecologically valid paradigms to understand psychiatric disorders, refer to Scholl and Klein-Flügge, 2018).

Thanks to the neuroscientific tools, neurological representations of the brain and neural activity can be generated. The purpose is straightforward, to have real-time insights into specific responses in the brain and nervous system, resulting from the presentation of a stimulus (Lim, 2018). Especially, eye-tracking technique provides a relatively low-cost and sensitive indicator for initial orienting, shift, and maintenance of attention (Caseras et al., 2007; Kou et al., 2016; Wolf et al., 2021). Since eye-movements provide realistic evidence of where participants are likely to look at Bird et al. (2012); Morii and Sakagami (2015); van der Laan et al. (2015); Wolf et al. (2018, 2019), this might be a valid reason why some research groups choose precisely the eye-tracking technique to study attentional biases among individuals with body dysmorphic disorder (BDD) (Kou et al., 2016), which is characterized by repetitive behaviors and/or mental acts occurring in response to preoccupations with perceived defects or flaws in physical appearance (American Psychiatric Association, 2013). For instance, to explore attentional bias toward body-related pictures among females with weight dissatisfaction Gao and colleagues employed the eye-movement technique. The researchers reported an orienting-maintenance pattern of attention toward fatness-related pictures among weight-dissatisfied women, implying that this group preferentially attended to body-related/fatness-related stimuli (Gao et al., 2012). In addition, results presented by Greenberg and her team also suggest that individuals with BDD “*overfocus on negative attributes*” (Greenberg et al., 2014). “*While current treatments generally show moderate effectiveness in the short-term, those with BDD can have high relapse rates, as they still* “*see” their flaws or defects”* (Beilharz et al., 2018). Therefore, elucidating the role of attention (e.g., negative/positive bias) may help to identify risk and maintenance factors among BDD patients (Grocholewski et al., 2012; Greenberg et al., 2014; Kollei et al., 2017). For example, in 2018, Beilharz and her colleagues proposed a visual training program (encompassing basic visual processing, face and emotion recognition, and self-perception), which has been designed to remediate visual abnormalities and reduce symptom severity among individuals with BDD (Beilharz et al., 2018).

In another elegant experiment, supported with the eye-tracking methodology, Toh and colleagues investigated facial affect recognition in BDD and obsessive-compulsive disorder patients. Relative to OCD patients and HCs, patients with BDD demonstrated (i) significantly poorer facial affect perception (misidentifying emotional expressions as angry, i.e., BDD participants misinterpreted more faces as angry in comparison to OCD patients and HCs); (ii) atypical scanning strategy characterized by significantly more blinks, fewer but longer fixations, and less visual attention devoted to the salient facial features (Toh et al., 2015). With this in mind, in the future, researchers may consider developing interventions that will reduce distress for individuals with high body image dissatisfaction (for future directions for positive body image research refer to Halliwell, 2015).

**FIGURE 2:**
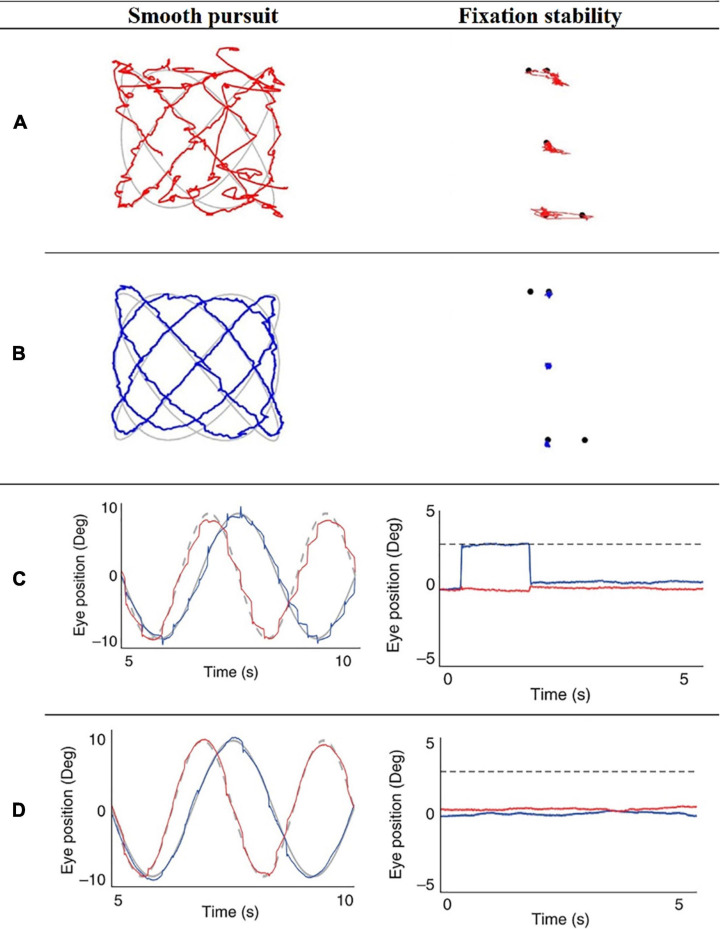
Ilustrative eye-movement recordings of pursuit and fixation stability tests of schizophrenia (SZ) patients and healthy controls (HC), obtained from Benson et al. (2012) and Morita et al. (2017). Importantly, the reader should bear in mind that not every participant will exhibit “normal/abnormal” eye-movements on every trial nor on each task. Illustrative Lissajous pursuit and fixation stability tests of SZ patient **(A)** and HC **(B)**, obtained from Benson et al. (2012). Representative eye-movement recordings of a patient with SZ **(C)** and one HC **(D)**, during the smooth pursuit eye movement test (fast Lissajous paradigm) and the fixation stability test (far distractor paradigm), obtained from Morita et al. (2017).

The scientific community is already witnessing a considerable rise in gaze metrics models targeting to unveil one’s values (goals, tradeoffs, objectives) and beliefs (facts, opinions, uncertainties), tapping into unconscious processes governed by individuals’ internal state (homeostasis). Clinicians have actively grasped the technological advances in vision science, demonstrating characteristic eye-movement distortions in patients with psychiatric disorders or individuals being potentially at risk of them (kindly refer to an illustrative example in Figure 2). An extensive number of clinical findings reported abnormal gaze parameters among clinical populations (e.g., prolongation of saccadic latency, abnormal smooth pursuit, ocular drift (glissades), square wave jerks, and impaired vestibulo-ocular reflex) (see Silverstein et al., 2015).

A great number of the clinical results (especially in the schizophrenia research domain) refers to now-classic paradigms, i.e., *smooth pursuit test* (where participants are required to track a moving target with their gaze) and *fixation stability test* (where participants are asked to maintain their gaze on a defined fixation point). Both paradigms are known for their overwhelming evidence in the scientific literature (Holzman et al., 1973, 1974, 1980; Holzman, 1985; Koychev et al., 2011), and are highly important to study attentional deficits. For example, it has been reported that SZ patients (and their biological family members) perform the smooth pursuit test inaccurately. Moreover, gaze maintenance on a single target (fixation) is unsteady among SZ patients. Besides, their first-degree relatives have been reported to be deficient in fixation maintenance as well (Benson et al., 2012).

**FIGURE 3:**
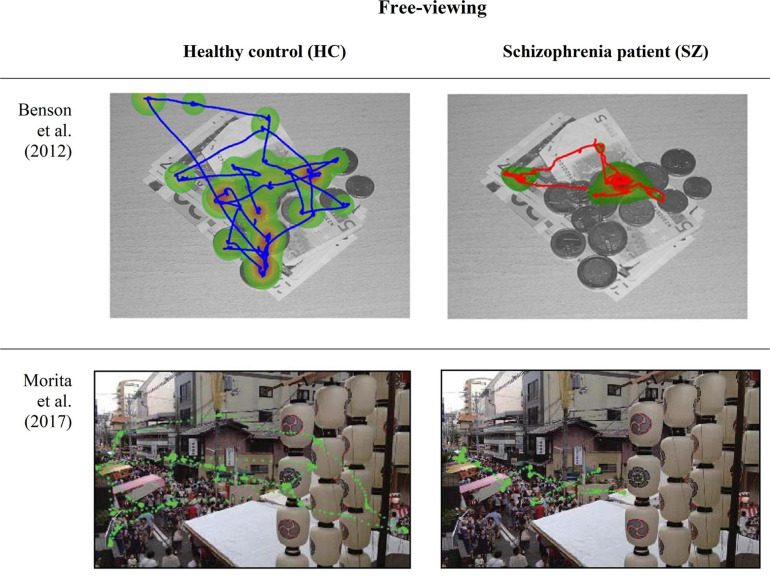
Eye-movement recordings of a patients with schizophrenia (SZ) and healthy controls (HC) during a free-viewing paradigm, obtained from Benson et al. (2012) and Morita et al. (2017).

To disclose one’s exploratory eye movement behavior, free-viewing paradigms are also widely applied in clinical research domain (Kogata and Iidaka, 2018; van Renswoude et al., 2018; Lakhlifi et al., 2020) (Figure 3). Abnormalities in visual scanning have been reported to afford an impressive accuracy of specificity and sensitivity. First, by demonstrating usefulness in quantitative scoring and sensitive detection of cognitive impairments and next, by tapping the subtype of mental illness (Beedie et al., 2011; Li et al., 2016, 2020a; Oyama et al., 2019).

Although now-classic (i.e., smooth pursuit and fixation stability) and free-viewing tests are highly visible in clinical experiments as routine screenings, simple and sufficiently short real-life decision-making paradigms supported with an eye-tracking technology, are scarcely being applied in a form of a screening tool for the early detection of cognitive decline (and related with it disorders). It is not in the scope of our article to list the benefits of an early diagnosis; however, we find it crucial to mention that it can give individuals at risk the opportunity to benefit from symptomatic treatments (Barnett et al., 2014, cited in Oyama et al., 2019). Additionally, the early detection of cognitive impairments can increase the efficiency of patient recruitment for clinical trials of drug development, which has shifted to focus on the early stage of some diseases (Graham et al., 2017; Carl et al., 2020).

Hence, to address the investigation on how clinical populations approach real-life functioning tasks, new research questions require analysis of decision-making paradigms that may provide informative attentional scan-paths and explorative eye-movements, beyond the quantification of fixations and saccades (Wolf et al., 2021). Furthermore, since decision-making paradigms fundamentally relate to the individual’s homeostatic processing, it seems straightforward to actively translate them into clinically performed experiments, supported with non-invasive eye-tracking devices.

## Translational Practice

Reflecting cognitive states of the viewer, eye-movement measurements have been reported to find potential application in the translational clinical practices. For instance, in the form of biomarkers, they may be used to identify individuals at risk, allowing efficient management of the process of early intervention. Therefore, in this part or the article, we anticipate how the knowledge from decision-making (information processing) paradigms that implement eye-tracking technology, can provide a beneficial platform for economists, psychiatrists, neurologists, and social and clinical psychologists to develop a common language for studying context-laden behavior in psychiatric disorders (Paulus, 2007). Such an approach may broaden current knowledge of mechanisms, which underlie illnesses and maladies (e.g., eating disorder, obsessive-compulsive personality disorder, pathological gambling) characterized by individuals’ inability to control their consumption practices (Javor et al., 2013).

### Misconception of Integrating Neuromarketing Into Clinical Experiments

It is important to solve the misconception of integrating neuromarketing into clinical experiments. In neuromarketing research, physiological and neuroscientific methods are being actively used to study consumer behavior to better understand the underlying neurobiology of psychological phenomena of decision-making processes, as well as to provide a more comprehensive assessment of the efficacy of marketing strategies such as advertising campaign planning or product positioning (for an excellent description on the most pertinent neuroscientific methods recommended for neuromarketing, kindly refer to Lim, 2018). Thus, neuromarketing may enclose a two-way interpretation of potential application: (*1*). *scientific*, focusing on consumer’s decision-making (action selection, from a set of available options) and (*2*). *commercial (for-profit)*, requiring scientific findings in order to establish the most profitable business strategies for consumers to purchase a certain product (Gidlöf et al., 2013, 2017; for a comprehensive debate on neuromarketing and consumer neuroscience, see Javor et al., 2013).

In recent years, due to sensational journalism, concerns related to subliminal advertising as a form of external purchase manipulations have emerged (for an insightful discussion on “Consumer Surveillance and Ethical Concerns” see Nemorin and Gandy, 2017). These misconceptions (i.e., scientifically unsupported controversial anecdotes) influence the academic efficacy and practical utility of neuroscientific measurement techniques in understanding the human decision-making processes (Pop et al., 2014; Thomas et al., 2016; Lim, 2018). Although Pop et al. (2014) clearly stated that *“One of the most important challenges for companies who offer neuromarketing services is to stick to ethical principles when performing the investigations. This is an obligation they have both toward the beneficiaries – the companies providing products or services – and toward their consumers as well (…),”* the dispute between the advocates and critics of neuromarketing remains present until today (Foscht and Swoboda, 2011; Pop et al., 2014).

Not to distract this highly important discussion, consumer neuroscience (that enriches understanding of consumer psychology and behavior) and neuroeconomics (that refers to sensemaking of economic problems through the analysis of neural correlates of decision making) should be studied, among healthy controls and clinical populations (Rahman et al., 2001; Nemorin and Gandy, 2017; Wolf et al., 2021). The decisive advantage is that with the support of neuromarketing and its neuroscientific methods the *reasons behind individuals’ decisions* can be investigated (Foscht and Swoboda, 2011).

Translational practices (of behavioral economics frameworks) have been mentioned to be useful to evaluate the dysregulation of reward-oriented behavior (Kobayashi et al., 2019). For example, patients with orbitofrontal cortex (OFC) damage have been reported to make poor decisions about day-to-day activities and engage in risk-taking behaviors (Bechara et al., 1994; Shiv et al., 2005; Floden et al., 2008), which may have negative consequences for their wellbeing and overall mental health (Rahman et al., 2001). Therefore, carrying out decision-making paradigms may help licensed therapists to validate the therapy directed toward patients with impulsive-compulsive disorders, such as for example pathological gamblers (frequently reported among individuals with Parkinson’s disease, frontotemporal dementia, and Huntington’s disease) and compulsive buyers (characterized with impulse control disorder, which co-occurs with depression) (De Marchi et al., 1998; Javor et al., 2013; Kalkhoven et al., 2014). In essence, neuromarketing studies, conducted in accordance with the Ethical Code of Conduct issued by the Neuromarketing Science and Business Association, would be of significant benefit to the general social progress (Pop et al., 2014).

### Eye-Trackers Offset Traditional Self-Reporting Assessments

We also need to address that in clinical research, mental disorders are mainly diagnosed through observations made by highly specialized psychiatrists that are based on a patient’s overall behavior and self-reports. This means that for a patient to receive a diagnosis of a mental disorder, standardized criteria such as alterations in behavioral and cognitive development, laid out by the Diagnostic and Statistical Manual of Mental Disorders (DSM-V) (American Psychiatric Association, 2013), must be visible to the clinician. However, similarly as in marketing research, traditionally used subjective tools (e.g., rating scales that allow respondents to provide nuanced answers, questionnaires, and tests) are characterized with well-known limitations (Connors et al., 2016; Bell et al., 2018), lacking credibility due to self-report biases that are present when responding to sensitive questions related to addictive behaviors and criminal or sexual experiences (Tourangeau and Yan, 2007). By offsetting weaknesses associated with traditional self-reporting assessments that are usually designed for one disorder or limited in their examination of phenomenological features, eye-tracking generates considerable interest in terms of consumer research not as a complementary but as a standing-alone technology, revealing one’s moment-by-moment internal state (homeostasis) (Ernst and Paulus, 2005; Paulus, 2007; Wolf et al., 2021).

Not restricted to economic disciplines only, the eye-tracking methodology represents a category of interdisciplinary research instrumentation that has successfully intermingled with various research questions/tasks and exercised human eye-behavior with numerous stimuli categories (geometrical figures, computerized and real human faces, naturalistic food images, haptic and pictorial illusions as well as advertisement videos). These compelling arguments stand for eye-tracking technology being actively used in interdisciplinary laboratories, generating behavioral experimental paradigms that integrate contributions from psychology, philosophy, and affective and computer sciences (Borji et al., 2013; Spinks and Mortimer, 2016; Vu et al., 2016; Gidlöf et al., 2017; Wolf et al., 2018; Zommara et al., 2018; Vriens et al., 2020).

Future paradigms, which incorporate a decisional context (homeostatic regulation), may generate findings of significant importance to all behavioral sciences (including medical sciences such as neurology and psychiatry). Although the current trend in clinical research does not aim to replace the diagnosis criteria that rely on clinical observations and self-report, recent studies started to test the possibility of gaze metrics to distinguish patients suffering from mental disorders from healthy participants, hoping for a successful diagnosing tool, and those potentially at risk, aiming for an early intervention chance (Benson et al., 2012).

Bridging decision-making science with medical science may support clinical interventions and contribute to health services research agenda, to improve population health outcomes. Ideally, scientists develop cognitively informative paradigms, aiming to understand behavior among HC, which can be then applied to individuals suffering from mental illnesses, in order to identify biomarkers that point to one’s brain integrity (mechanism of information processing that requires a synchronized activity of lower and higher-order brain structures). Clinical paradigms, however, tend not to implement higher-order cognitive components related to decision-making paradigms, based on neurobiologically informed economic theories and mathematical choice psychology. Thus, a gap is being created where the translational approach of decision-making paradigms, that involves testing of the potential biomarkers in clinical trials, is scarcely reached. If not cued by cognitive scientists, eye-tracking technology may remain a theoretical recommendation for clinical practices (Wolf et al., 2021).

## Discussion

To capture and investigate an interplay between cognition (and its deficits) and eye-movements, information-processing paradigms that reveal one’s gaze-patterns are needed. Following the global trend of the so-called *digital pharma* (or *beyond the pill*) strategy (Rutkowski et al., 2020) there is a need for technologies that support early diagnostics for cognitive interventions and monitor individual’s mental wellbeing. In our opinion, eye-movement measurements come in as a relatively low-cost measurable indicator (biomarker) of one’s homeostatic (mental) state. It has been reported that cognitive dysfunctions detected through gaze analysis may indicate or even predict mental disease processes (Fujioka et al., 2016; Almubark et al., 2020; Wolf et al., 2021). Moreover, studies of significant importance have produced preliminary findings that show that gaze-metrics parameters such as fixations (their location, number and duration), saccades (their number and amplitudes) and the scan-path length, are abnormal in a great number of neurological diseases (Beedie et al., 2011; Benson et al., 2012; Türkan et al., 2016; Li et al., 2020b; Morita et al., 2020).

However, cognitively informative paradigms are needed to draw further implications for medical experts and clarify aspects of visual impairments that manifest among clinical populations. Such paradigms may provide additional knowledge regarding attention, engagement and memory retention, all together combined as the foundation of an undertaken decision (Scholl and Klein-Flügge, 2018). When taking into consideration that approximately 95% of human decision-making processes happen at the subconscious level (Zaltman, 2003; Pop et al., 2014; Nyoni and Bonga, 2017), neuroscience opens the possibility of getting closer to the invisible part of neuronal connections, overcoming limitations related to self-reported methodologies (Connors et al., 2016; Bell et al., 2018).

Since numerous clinical works call for an etiology-based diagnosis (i.e., to understand the brain processes of individuals, who suffer from mental disorders), the development of reliable biomarkers (improving the diagnosis, identifying at-risk-individuals, and providing novel targets for therapeutic interventions), is not only in highly demand but it is the main purpose of modern clinical research (Kim et al., 2011; Daglas et al., 2015; Yahata et al., 2017; Wolf et al., 2021).

Eye-behavior tests that discriminate clinical cases from control subjects, have mounted in recent years. Shiino and colleagues reported significant differences between subjects with ASD and SZ in 5 selected eye-movement characteristics that were obtained from free-viewing and smooth pursuit tests (Shiino et al., 2020). Some other research groups modernized the clinical development process by integrating digital methods, based on machine learning (ML) approach (Benson et al., 2012; Tseng et al., 2013). For example, Tseng et al. (2013) extracted quantitative features from gaze data with the support of automated ML. Following this procedure, the authors were able to differentiate patients with attention deficit hyperactivity disorder (ADHD) and fetal alcohol spectrum disorder (FASD) with overlapping behavioral phenotypes from age-matched healthy participants (Tseng et al., 2013). Therefore, ML techniques extracting disorder-specific features for an automatic classification should be considered in prospective clinical applications (Huys et al., 2016; Yahata et al., 2017).

It is important to mention that eye-trackers can be utilized as a new intervention training tool for exposure therapies or attention redirection training. In the context of obsessive-compulsive disorder (OCD), Bradley and colleagues reported that eye-tracking, attentional control, and severity of attentional bias can indicate the therapeutic progress of implemented treatment plan, e.g., exposure response prevention — ERP (Bradley et al., 2016). Therefore, monitoring obsessions, compulsions, and attentional biases in a real-life context may determine therapy outcomes more accurately.

An implementation of real-life inspired paradigms related to general as well as social cognitive impairments will allow a better understanding of the homeostatic processing abnormalities among clinical populations (Ernst and Paulus, 2005; Paulus, 2007; Billeke and Aboitiz, 2013). At the same time, the support of eye-tracking technology will help to objectively illustrate and measure how and when information processing goes away from the expected course. For comprehensive and interdisciplinary models to be built in the future, experimental paradigms resembling real-life activities should be conducted, to have a clearer and disorder-specific picture of how converging as well as diverging tasks are processed inside patient’s mind and interpreted by her eyes.

### Limitations

Since gaze metrics, gathered from visual information processing tasks, have recently started to support the development of non-invasive and relatively inexpensive biological markers in the clinical research domain, the presented article aimed to draw readers attention to one particular neuroscientific tool, namely the eye-tracking technology. Other neuroscientific methods were omitted due to the intentionally narrowed scope of this article.

Presented work is likely generalizable to journal articles and systematic reviews acquired through the Kyushu University Open Access Policy. Furthermore, to refrain from any unintended bias (caused by the authors’ research background), while addressing the aim of the perspective article, AW and KU have included a great number of articles, which relate to now-classic and modern clinical studies. The protocol was drafted using PRISMA guidelines, revised by the authors and lab members to solicit additional feedback. Articles published between 2010 and 2020, reporting cognitive impairments or abnormal eye-movements patterns among clinical populations, were identified by Mendeley and PubMed searches (search terms were: “eye-tracking”, “shopping task,” “cognitive deficits,” “clinical application,” and “cognitive impairments”). Reference lists of all in-scope articles have been additionally screened for relevant publications. Notably, to interlock the trend of decision-making paradigms, non-clinical open access articles were included in this work as well.

Finally, despite the absence of empirical data, best efforts have been undertaken to produce an objective, academic article that refrains from scientifically unsupported sensational claims concerning neuromarketing and its neuroscientific methods. It is hoped that the gathered examples will propel a greater interest and insightful research in consumer behavior and neuroeconomics, among individuals with mental disorders.

## Data Availability Statement

The original contributions presented in the study are included in the article/supplementary material, further inquiries can be directed to the corresponding author.

## Author Contributions

AW wrote the manuscript with a critical revision from KU. Both authors contributed to the article and approved the submitted version.

## Conflict of Interest

The authors declare that the research was conducted in the absence of any commercial or financial relationships that could be construed as a potential conflict of interest.
